# Vitamin D and Cardiovascular Disease: The “Good”, the “Bad”, and the “Unknown”

**DOI:** 10.31083/RCM40001

**Published:** 2025-10-27

**Authors:** Domenico Mario Giamundo, Matteo Morello, Paola Pastena, Marco Bernardi, Luigi Spadafora, Stefano Cacciatore, Francesco Perone, Giuseppe Caminiti, Pierre Sabouret, Arturo Cesaro, Francesca Maria Di Muro, Michele Golino

**Affiliations:** ^1^Department of Systems Medicine, University Tor Vergata, 00133 Rome, Italy; ^2^Robert M. Berne Cardiovascular Research Center and Department of Medicine, University of Virginia, Charlottesville, VA 22908, USA; ^3^Department of Molecular and Translational Medicine, University of Brescia, 25121 Brescia, Italy; ^4^Division of Cardiology, Department of Pediatrics, Columbia University Irving Medical Center, New York, NY 10032, USA; ^5^Department of Medico-Surgical Sciences and Biotechnologies, Sapienza University, 00185 Latina, Italy; ^6^Department of Geriatrics, Orthopedics, and Rheumatology, Università Cattolica del Sacro Cuore, 00168 Rome, Italy; ^7^Fondazione Policlinico Universitario “Agostino Gemelli” IRCCS, 00168 Rome, Italy; ^8^Cardiac Rehabilitation Unit, Rehabilitation Clinic “Villa delle Magnolie”, Castel Morrone, 81020 Caserta, Italy; ^9^Department of Human Science and Promotion of Quality of Life, San Raffaele Open University, 00166 Rome, Italy; ^10^Cardiology Rehabilitation Unit, IRCCS San Raffaele, 00166 Rome, Italy; ^11^Heart Institute, Boulevard de l'Hôpital, ACTION Study Group-CHU Pitié-Salpétrière Paris, 75013 Paris, France; ^12^Collège National des Cardiologues Français (CNCF), 75014 Paris, France; ^13^Department of Translational Medical Sciences, University of Campania “Luigi Vanvitelli”, 80138 Naples, Italy; ^14^Division of Cardiology, A.O.R.N. “Sant’Anna e San Sebastiano”, 81100 Caserta, Italy; ^15^Icahn School of Medicine at Mount Sinai, New York, NY 10029, USA; ^16^Cardiology Unit, Cardiovascular and Thoracic Department, University Hospital San Giovanni di Dio e Ruggi d’Aragona, 84131 Salerno, Italy; ^17^Pauley Heart Center, Virginia Commonwealth University, Richmond, VA 23298, USA

**Keywords:** vitamin D deficiency, cardiovascular diseases, hypertension, heart failure, atherosclerosis

## Abstract

Vitamin D is a key regulator of calcium and phosphorus homeostasis; meanwhile, the dietary absence of vitamin D represents the most common nutritional deficiency worldwide. The discovery of vitamin D receptors and conversion enzymes within the cardiovascular system has fueled growing interest in the potential roles of vitamin D beyond bone health. Indeed, preclinical studies have suggested that vitamin D might regulate vascular tone and exert antifibrotic and anti-remodeling effects on the myocardium. Furthermore, a deficit in vitamin D has been associated with an increased risk of hypertension, atherosclerosis, and heart failure. These findings have prompted several interventional studies to investigate whether vitamin D supplementation can mitigate cardiovascular risk. However, current evidence regarding the cardiovascular benefits of vitamin D intake remains inconsistent and inconclusive. This review aims to provide a comprehensive overview of the “good”, the “bad”, and the “unknown” aspects of the relationship between vitamin D and cardiovascular disease.

## 1. Introduction

Despite significant advances in the prevention and treatment of cardiovascular disease (CVD), one person in the United States dies from heart disease or stroke every 34 seconds [1, 2]. This alarming statistic underscores the urgent need to identify novel, modifiable risk factors beyond traditional targets such as hypertension, hyperlipidemia, or diabetes. Among emerging candidates, Vitamin D, a fat-soluble nutrient historically associated with bone homeostasis, has garnered increasing attention for its potential role in CVD health.

Originally discovered in the context of rickets, vitamin D has long been recognized as a cornerstone of calcium and phosphate metabolism [3]. However, the subsequent identification of vitamin D receptors (VDRs) and associated metabolic enzymes widely expressed across the cardiovascular tissues, including cardiomyocytes, vascular smooth muscle cells, and endothelial tissue, have led to the hypothesis that vitamin D may exert pleiotropic effects on key pathophysiological mechanisms such as vascular tone regulation, myocardial remodeling, inflammation, fibrosis, and atherogenesis [4, 5, 6].

Vitamin D deficiency, or hypovitaminosis D, is still considered the most prevalent nutritional deficiency worldwide. It affects about one billion people, with high prevalence in older adults and those with limited sun exposure or darker skin pigmentation [7, 8]. In addition to its well-established role in bone health and calcium and phosphorus homeostasis, low vitamin D levels may increase the risk of several CVDs, such as hypertension, coronary artery disease, and heart failure (HF) [9, 10, 11]. This knowledge has raised the possibility that vitamin D could represent a low-cost and widely accessible tool for cardiovascular risk reduction.

Several interventional studies have reported conflicting results and no consistent cardiovascular benefits. Even a large meta-analysis has failed to establish a definitive role for vitamin D supplementation in cardiovascular risk reduction [12]. In this review, we examined the “*good*”, the “*bad*”, and the “*unknown*” of the relationship between vitamin D and cardiovascular health.

## 2. The Biological Pathway of Vitamin D: From Skin to Cell Nucleus

**Fig. 1.**
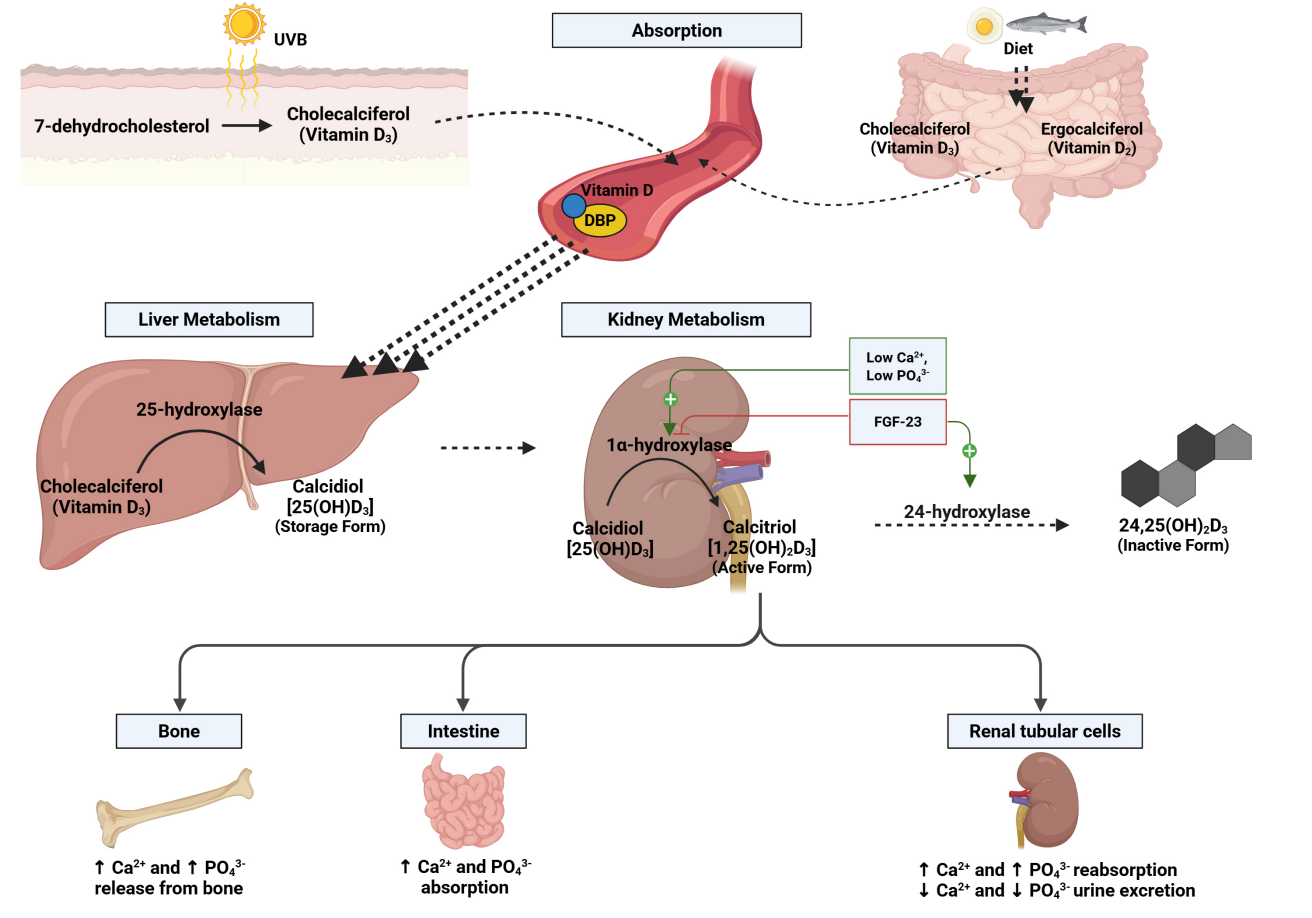
**Vitamin D metabolism pathway**. Vitamin D metabolism begins with its production in the skin (under ultraviolet B (UVB) light, as cholecalciferol or Vitamin D3) or its intake from food (as cholecalciferol, Vitamin D3, or ergocalciferol, Vitamin D2). After absorption, Vitamin D binds to a transport protein (DBP) and travels to the liver. There, it is converted into calcidiol [25(OH)D_3_], the storage form, by the enzyme 25-hydroxylase. In the kidneys, calcidiol is converted into calcitriol [1,25(OH)_2_D_3_], the active form, through 1α-hydroxylase. This step is positively controlled by calcium and phosphate levels and negatively influenced by fibroblast growth factor (FGF)-23, which also influences the inactivation into [24,25(OH)_2_D_3_] by 24-hydroxylase. Calcitriol regulates calcium and phosphate in the body by increasing their release from bones (through increased osteoclast activity), improving absorption in the gut, and reducing loss in urine (increased reabsorption in the proximal renal tubule). Created in BioRender (https://BioRender.com/3cqhys3). Golino, M. (2025).

Vitamin D exists in two dietary forms: D3 (cholecalciferol) and D2 (ergocalciferol), which differ slightly in structure [13]. Vitamin D metabolism begins from the synthesis of 7-dehydrocholesterol, which undergoes two sequential hydroxylations: the first, by hepatic 25-hydroxylase, produces 25-hydroxyvitamin D (25(OH)D), and the second, by 1-α-hydroxylase in the kidneys, produces 1,25-dihydroxyvitamin D (1,25(OH)_2_D), also known as “calcitriol” which is the biologically active form (Fig. 1).

Both circulating 25(OH)D and 1,25(OH)_2_D are mainly bound to vitamin D binding protein (DBP) and albumin; however, the half-life of circulating 25(OH)D (10–20 days) is higher than that of 1,25(OH)_2_D (10–20 hours), due to the higher affinity for DBP of the former [14, 15]. Circulating 1,25(OH)_2_D is tightly regulated by parathyroid hormone (PTH) and fibroblast growth factor-23 (FGF-23) to maintain plasma calcium and phosphate within their physiological ranges [7, 16]. Calcitriol binds the vitamin D receptor (VDR), inducing a conformational change that leads to hetero-dimerization with the retinoid X receptor (RXR) and translocation of this complex into the nucleus, where it binds to the promoter region of more than 200 target genes [17]. Although 25(OH)D is the preferred biomarker of vitamin D level due to its longer half-life, there is still no universal consensus on the optimal threshold values. Most guidelines define deficiency as a serum concentration below 30 nmol/L. In contrast, sufficiency is variably defined, ranging from >50 nmol/L as recommended by the European Society for Clinical and Economic Aspects of Osteoporosis [18] to >75 nmol/L according to the Endocrine Society [7]. To maintain adequate levels in the absence of enough sunlight exposure, daily vitamin D intake is generally recommended to range from 600 to 2000 international units [7].

## 3. The “Good”

Vitamin D plays an active role in cardiovascular physiology, primarily mediated by the expression of its receptors and activating enzymes in cardiomyocytes, endothelial cells, and vascular smooth muscle cells [19]. Preclinical studies have shown that VDR-null mice exhibit increased left ventricular mass, elevated atrial natriuretic peptide levels, and dysregulation of cardiac metalloproteinases and fibroblasts. These alterations promote fibrotic extracellular matrix deposition, leading to ventricular dilatation and impaired electromechanical coupling [20, 21, 22, 23, 24, 25].

**Fig. 2.**
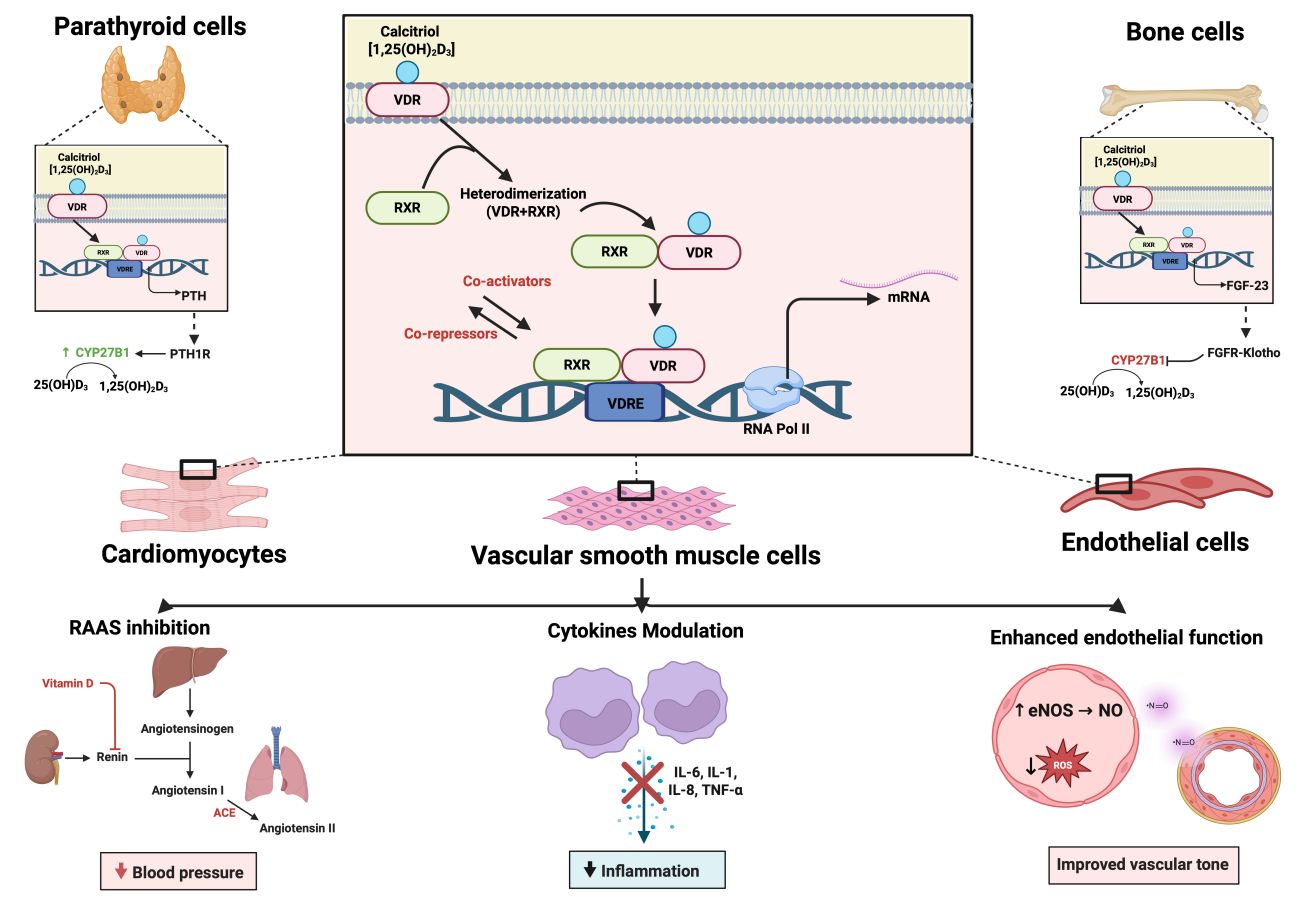
**Mechanisms of Vitamin D on the cardiovascular system**. The active form of Vitamin D (calcitriol or 1,25(OH)_2_D_3_) binds to the Vitamin D receptor (VDR), which heterodimerizes with the retinoid X receptor (RXR). This complex binds to vitamin D response elements (VDREs) in target genes, modulating transcription via co-activators and co-repressors. The effects are cell-type specific: in parathyroid cells, Vitamin D downregulates parathyroid hormone (PTH) expression; in bone cells, it promotes FGF-23 production; and in cardiovascular cells-including cardiomyocytes, vascular smooth muscle cells, and endothelial cells-it mediates beneficial effects via: inhibition of the renin-angiotensin-aldosterone system (RAAS), downregulation of pro-inflammatory cytokines (IL-6, IL-1, IL-8, TNF-α), and enhancement of endothelial nitric oxide production and vascular tone. These actions collectively contribute to reduced blood pressure, decreased inflammation, and improved vascular function. Created in BioRender (https://BioRender.com/t23l764). Pastena, P. (2025).

In endothelial cells, VDR activation regulates vascular endothelial growth factor expression, influences calcium influx, and modulates the vascular endothelium-dependent tone [26, 27]. In VDR-deficient mice, endothelial nitric oxide (NO) synthase is reduced by more than 50%, and acetylcholine-induced aortic relaxation is considerably impaired [28, 29]. The increased renin expression and renin-angiotensin-aldosterone system (RAAS) activation have been suggested as additional mechanisms, as observed in Fig. 2 [30]. Therefore, in hypertensive rats, chronic treatment with 1,25(OH)_2_D showed to reduce reactive oxygen species (ROS) levels and cyclooxygenase-1 (COX-1) expression with beneficial effects on blood pressure [31].

Vitamin D also exerts significant anti-inflammatory effects, modulating both innate and adaptive immune responses. It suppresses proinflammatory cytokines such as interleukin (IL)-6, tumor necrosis factor-alpha (TNF-α), and IL-23 while promoting the release of anti-inflammatory mediators including IL-10 and IL-4 [19, 32, 33]. Notably, both 1,25-dihydroxyvitamin D [1,25(OH)_2_D] and 25-hydroxyvitamin D [25(OH)D] act through mitogen-activated protein kinase phosphatase-1—a signaling pathway activated in monocytes and macrophages—to inhibit the production of TNF-α and IL-6 [34]. *In vitro* studies have also demonstrated that 1,25(OH)_2_D attenuates Toll-like receptor (TLR)-mediated inflammatory responses and downregulates the production of proinflammatory microRNA-155 in macrophages [35]. Active vitamin D also promotes macrophage polarization toward an anti-inflammatory M2 phenotype, as shown by increases in CD206 and IL-10 expression and enhanced M2 markers in both cell culture and animal studies [36]. Additionally, vitamin D/VDR directly suppresses NOD-like receptor family pyrin domain containing 3 (NLRP3) inflammasome activation by binding NLRP3 and preventing BRCC3-mediated deubiquitination, thereby inhibiting IL-1β secretion and pyroptosis [37]. VDR activation also represses NF‑κB signalling and upregulates SOCS1, providing negative feedback control of TLR4-mediated signaling [38]. Vitamin D has further been shown to modulate adaptive immunity by inhibiting pro-inflammatory T-cell and dendritic cell differentiation while supporting T-regulator and natural killer (NK) cell function [39]. In a double knockout mouse model lacking both the VDR and IL-10, accelerated progression of inflammatory bowel disease was observed, accompanied by increased TNF-α expression. Administration of 1,25(OH)_2_D combined with a high-calcium diet significantly reduced TNF-α levels and attenuated disease severity [40]. Together, these pathways underscore the multilevel role of vitamin D in immune homeostasis and its potential to limit chronic systemic and cardiovascular inflammation.

Beyond its broad anti-inflammatory effects, vitamin D plays a key role in modulating the pathogenesis of atherosclerosis. It influences monocyte activity and the regulation of matrix metalloproteinases (MMPs). Specifically, vitamin D has been shown to reduce the expression of TNF-α, IL-6, IL-1, and IL-8 in isolated blood monocytes [6, 41]. Suppression of IL-6 contributes to decreased C-reactive protein (CRP) levels, an acute-phase reactant and a well-established predictor of atherosclerotic disease and cardiovascular events [42].

Nakagawa *et al*. [43] demonstrated that 1,25(OH)_2_D downregulates MMP-2 and MMP-9 expression in cultured cells, stabilizing atherosclerotic plaques and reducing the risk of rupture, thrombosis, and lumen obstruction. Vitamin D also reduces cholesterol accumulation in macrophages and inhibits low-density lipoprotein (LDL) uptake within atheromatous plaques [44]. Furthermore, it modulates thrombogenic activity by regulating thrombomodulin and tissue factor expression in monocytes, thereby affecting platelet aggregation and coagulation potential [45].

Vitamin D improves endothelial function by upregulating endothelial NO synthase (eNOS) through phosphoinositide 3-kinase/protein kinase B (PI3K/AKT)-dependent pathways, enhancing NO production and reducing endothelial oxidative stress [46]. Furthermore, vitamin D suppresses NF-κB-mediated expression of endothelial adhesion molecules such as vascular cell adhesion molecule 1 (VCAM-1), intercellular adhesion molecule 1 (ICAM-1), and E-selectin, thereby limiting monocyte adhesion and early plaque formation [47]. Vitamin D has also been shown to inhibit vascular smooth muscle cell proliferation and migration, thereby reducing neoinitimal hyperplasia and plaque progression [48]. Vitamin D also exerts atheroprotective effects by inhibiting NLRP3-inflammasome activity within endothelial cells and macrophages, leading to reduced IL‑1β and IL‑18 release within plaques [37].

In apolipoprotein E-deficient mouse models, active vitamin D administration reduced the number of atherosclerotic lesions, decreased macrophage infiltration, and limited CD4+ T-cell accumulation in the aortic sinus. Oral calcitriol further attenuated atherosclerosis by promoting the induction of regulatory T cells and immature dendritic cells with tolerogenic properties [49].

Experimental evidence also indicates that vitamin D enhances insulin secretion and sensitivity by modulating both pancreatic and inflammatory pathways [50]. The discovery of VDRs in pancreatic β-cells has fueled interest in the potential role of vitamin D deficiency in insulin resistance and type 2 diabetes [51, 52, 53, 54]. Calcitriol has been shown to influence insulin secretion by modulating voltage-dependent calcium channels, which are essential for insulin granule exocytosis. In healthy individuals, VDR expression is particularly enriched in pancreatic islets but is diminished in individuals with diabetes.

*In vitro* studies using rat insulinoma-derived β-cells demonstrated that calcitriol upregulates genes such as *Vdr*, *Gck* (glucokinase), and *Insrb* (insulin receptor beta), while leaving other genes unaffected. Moreover, VDR expression in human islets correlates positively with the expression of calcium-handling genes and is upregulated by agents such as rosiglitazone and dexamethasone but not by metformin or insulin. These findings support a mechanistic model in which vitamin D enhances β-cell function and insulin secretion through VDR- and calcium-dependent mechanisms, independent of phospholipase C activation [51].

These findings suggest that vitamin D may contribute to glycemic control via direct effects on pancreatic β-cells and exert broader cardiometabolic benefits through its anti-inflammatory, antiatherosclerotic, and immunomodulatory properties.

## 4. The “Bad”

The role of vitamin D in cardiovascular health is complex and, at times, controversial. While vitamin D deficiency is associated with adverse outcomes, excessive levels may also have harmful effects. One major concern involves the potential link between vitamin D supplementation and vascular calcification. Vascular calcification results from the deposition of calcium phosphate crystals within arterial walls, reducing arterial compliance and increasing cardiovascular risk. Preclinical studies have reported that supraphysiological vitamin D levels can induce vascular calcification in animal models [55, 56]. For instance, rats administered high doses of vitamin D showed significant arterial stiffness and aortic calcification [57]. Similarly, vitamin D and calcium supplementation promoted vascular calcification in pseudoxanthoma elasticum mouse models [58]. Notably, such vascular remodeling appeared reversible upon reduction of vitamin D levels [57]. Human data also support this association. Case reports and small cohort studies have described metastatic arterial calcifications and soft-tissue calcifications in patients with hypervitaminosis D, hypercalcemia, and extremely elevated 25(OH)D levels (e.g., >150 ng/mL) [59]. Vascular calcification has also been observed in patients receiving high-dose vitamin D or alendronate combined regimens [60]. Observational studies have identified a U- or J-shaped association between serum 25(OH)D concentrations and cardiovascular morbidity and mortality, suggesting that both low (<20 ng/mL) and high (>50–60 ng/mL) vitamin D levels are associated with increased risk [61]. However, a randomized controlled trial using daily vitamin D_3_ 400–10,000 IU for 3 years found no difference in development or progression of lower limb artery calcification, suggesting that vascular calcification may depend on individual vulnerability or metabolic context rather than supplement dose alone [62]. Despite these concerns, randomized trials investigating the effects of vitamin D supplementation on blood pressure and arterial stiffness have mainly yielded disappointing results. For instance, administration of high-dose cholecalciferol (15,000 International Units (IU)/day for 1 month) to obese hypertensive subjects resulted in a modest reduction in mean arterial pressure but also heightened angiotensin sensitivity and increased aldosterone secretion [63, 64]. Several meta-analyses have confirmed the lack of significant clinical benefit of vitamin D supplementation on vascular stiffness and endothelial function. In a review of 13 Randomized Controlled Trials (RCTs), Rodríguez *et al*. [65] reported nonsignificant reductions in pulse wave velocity and augmentation index (–0.1 m/s and –0.15, respectively; *p* = 0.17 and 0.08). Similar findings were observed by Joris and Mensink [66], and Stojanović and Radenković [67], who found no improvement in brachial artery flow-mediated dilation after vitamin D intake, across diverse populations.

Large-scale RCTs, including patients with pre-hypertension or stage I hypertension, further reinforced these findings. A six-month study comparing daily high-dose (4000 IU) versus low-dose (400 IU) cholecalciferol found no significant difference in 24-hour systolic blood pressure (–0.8 vs –1.6 mm Hg; *p* = 0.71) [68]. Likewise, an Austrian RCT of 188 hypertensive patients receiving 25(OH)D <30 ng/mL showed no antihypertensive effect with 2800 IU/day compared to placebo (–0.4 mm Hg; *p* = 0.712) [69]. These results are further supported by a comprehensive meta-analysis of 46 RCTs involving 4541 participants, which found no significant effect of vitamin D supplementation on systolic (–0.5 mm Hg; *p* = 0.27) or diastolic (0.2 mm Hg; *p* = 0.38) blood pressure [70]. Similarly, Qi *et al*. [71] evaluated 8 RCTs in non-chronic kidney disease (CKD) individuals with pre-hypertension or hypertension and found no significant effect of vitamin D supplementation on either systolic (–0.08 mm Hg; *p* = 0.2) or diastolic (0.09 mm Hg; *p* = 0.155) blood pressure compared to placebo.

Large-scale randomized controlled trials underscore this lack of clinical benefit (Table 1, Ref. [72, 73, 74]). VINDICATE (VItamiN D treatIng Patients with Chronic heArT failurE) [72], VITAL (VITamin D and omegA-3) [73], and VIDA (Vitamin D Assessment Study) [74], trials failed to demonstrate meaningful reductions in cardiovascular outcomes with vitamin D supplementation. The VINDICATE trial, which focused on patients with systolic HF, evaluated 4000 IU/day of vitamin D3 for 12 months. Although improvements in left ventricular structure and function were seen, no gains were observed in functional capacity, as measured by the six-minute walk test, suggesting limited clinical impact despite structural changes [72]. The VITAL trial [73] enrolled over 25,000 middle-aged and older adults and randomized them to receive vitamin D3 (2000 IU/day) and/or omega-3 fatty acids. After more than six years of follow-up, no significant reductions were observed in rates of myocardial infarction, stroke, or cardiovascular death when compared with placebo. In the ViDA trial conducted in New Zealand, participants received 100,000 IU/month of vitamin D3 or placebo for approximately three years. This regimen also did not reduce the incidence of cardiovascular events, including myocardial infarction, angina, or stroke [74].

**Table 1.** **Recent RCTs on vitamin D supplementation and cardiovascular outcomes**.

Study	Year	Study design	Population	Intervention	Outcomes	Main findings
VINDICATE (VitamIN D treatIng patients with Chronic heArT failurE) study [72]	2016	Randomized, double-blind, placebo-controlled trial	n = 229 adults with HFrEF and vit D deficiency	Daily vitamin D3 (4000 IU) vs. placebo for 12 months	Primary endpoint: 6MWT distance. Secondary endpoints: LVEF, left ventricular dimensions (LVEDD, LVESD), left ventricular volumes (LVEDV, LVESV), renal function, serum calcium, PTH levels	No improvement in 6MWT. ↑ LVEF by 6.1%, ↓ LV dimensions. Safe, no hypercalcemia or renal harm.
VITamin D and OmegA-3 TriaL (VITAL) [73]	2019	Randomized, double-blind, placebo-controlled trial	n = 25,871 adults, ≥50 (men)/≥55 (women), (including 5106 African Americans)	Daily vitamin D3 (2000 IU) vs. placebo for 5.3 years	Major cardiovascular events (myocardial infarction, stroke, cardiovascular mortality), total cancer incidence, cancer mortality, all-cause mortality	No reduction in major CVD (HR 0.97), total cancer (HR 0.96), or mortality. ↓ Cancer mortality after excluding first 2 years (HR 0.75).
Vitamin D Assessment (ViDA) study [74]	2020	Randomized, double-blind, placebo-controlled trial	n = 5110 adults 50–84 yrs	Monthly high-dose vitamin D3 (100,000 IU) vs. placebo for a median of 3.3 years	Primary endpoints: CVD, acute respiratory infections, fractures, falls, total cancer incidence. Secondary outcomes: statin persistence, lung function, BMD, arterial function.	No effect on CVD (HR 1.02), fractures, falls, or cancer. Improved statin adherence (HR 1.15; *p* = 0.02), better lung function in ever-smokers (+57 mL FEV1; *p* = 0.03), and enhanced arterial function in vitamin D–deficient individuals (*p* = 0.03).

Studies are ordered by year of publication. Abbreviations: 6MWT, 6-Minute Walk Test; BMD, Bone Mineral Density; CVD, Cardiovascular Disease; FEV1, Forced Expiratory Volume in 1 Second; HF, Heart Failure; HFrEF, Heart Failure with Reduced Ejection Fraction; HR, hazard ratio; LVEDD, Left Ventricular End-Diastolic Diameter; LVEDV, Left Ventricular End-Diastolic Volume; LVEF, Left Ventricular Ejection Fraction; LVESD, Left Ventricular End-Systolic Diameter; LVESV, Left Ventricular End-Systolic Volume; LVSD, Left Ventricular Systolic Dysfunction; PTH, Parathyroid Hormone; RCT, Randomized Controlled Trial; ViDA, Vitamin D Assessment study; ↑, Increase; ↓, Reduction.

## 5. The Unknown

A persistent uncertainty in the relationship between vitamin D–and cardiovascular disease lies in the inconsistency across the available evidence. Meta-analyses by Parker *et al*. [75], Zittermann *et al*. [76], and Gaksch *et al*. [77] report inverse associations between circulating vitamin D levels and cardiovascular risk or all-cause mortality. Parker *et al*. [75] found that individuals with the highest vitamin D levels had 43% lower odds of cardiometabolic disorders (odds ratio (OR) 0.57, 95% confidence interval (CI): 0.48–0.68); Zittermann *et al*. [76] observed a nonlinear reduction in all-cause mortality with optimal 25(OH)D concentrations around 75–87.5 nmol/L; and Gaksch *et al*. [77], using pooled individual data from over 26,000 participants, showed significantly higher mortality risk at levels below 30 nmol/L compared to the reference range of 75–100 nmol/L. However, these findings contrast sharply with the results from RCTs, which have not consistently demonstrated the clinical benefits of vitamin D supplementation. In particular, the meta-analysis by Barbarawi *et al*. [12] found no significant reduction in major adverse cardiovascular events among vitamin D-treated patients, and Bjelakovic *et al*. [78] reported only a minor all-cause mortality benefit, exclusively linked to vitamin D3 and not D2 or active analogs. These discrepancies may be attributed to methodological differences, confounding factors such as physical activity, sun exposure, comorbidities, and baseline 25(OH)D levels. Notably, neither VITAL [73] nor ViDA [74] stratified participants by baseline vitamin D status, which may have diluted any effect in individuals with profound deficiency.

In VITAL, for instance, only a small subset of ~500 participants had 25(OH)D levels below 25 nmol/L [73]. Additionally, ethnic disparities may contribute to inconsistent findings. For example, individuals with darker skin pigmentation often have lower serum 25(OH)D levels due to reduced cutaneous synthesis, yet the clinical relevance of this biochemical deficiency remains debated [79, 80]. In VITAL, over 20% of participants were African American, a group that tends to have lower vitamin D levels but may be less susceptible to its adverse skeletal or cardiovascular consequences, possibly due to differences in vitamin D-binding protein polymorphisms and tissue-level vitamin D responsiveness [81]. Thus, any potential benefit may be restricted to severely deficient individuals underrepresented in these trials. Future trials may need to stratify by ethnicity, baseline deficiency, and genetic polymorphisms to more accurately identify responders to vitamin D supplementation.

Vitamin D status is heavily influenced by non-nutritional variables, making causal inference complex. Physical activity strongly correlates with higher serum 25(OH)D levels, possibly through enhanced lipolysis and release from adipose stores [82, 83, 84, 85, 86]. However, this association may be exercise-specific: while continuous combination training increased serum vitamin D, endurance training did not show similar effects [85]. Sun exposure, the major endogenous source of vitamin D, introduces further bias. Individuals with higher outdoor activity levels not only have greater vitamin D production but also tend to have lower baseline cardiovascular risk, potentially confounding associations between vitamin D and CV outcomes. The heterogeneity in dosing regimens, supplementation duration, and study populations further limits comparability across trials and the generalizability of results. Many trials enrolled elderly, institutionalized, or comorbid individuals whose high disease burden may have masked subtle cardiovascular benefits from vitamin D repletion.

There is no consensus on optimal serum 25(OH)D thresholds for cardiovascular protection. The Institute of Medicine (IOM) recommends daily intakes of 400–800 IU primarily for skeletal health but notes that current evidence is insufficient to support recommendations for cardiovascular outcomes [87]. Meanwhile, others suggest that 1500–2000 IU/day may be necessary to maintain optimal serum levels (>30 ng/mL), especially in at-risk individuals or those at higher latitudes [88, 89]. However, a linear inverse relationship between 25(OH)D and CVD risk appears to plateau around 60 nmol/L, and higher levels do not confer further protection, raising concern about over-supplementation [7]. Preliminary evidence suggests that specific subgroups, such as patients with congestive HF, CKD, or poorly controlled diabetes, may derive modest, condition-specific benefits from vitamin D. In patients with advanced CKD and low 25(OH)D, supplementation was associated with reduced cardiovascular events [90]. In contrast, in patients with diabetes and low vitamin D status, a single high-dose administration improved systolic BP and B-type natriuretic peptide levels [91]. Yet overall, the long-term cardiovascular effects of vitamin D remain unresolved. Furthermore, several studies suggest that the effects of vitamin D may vary depending on specific patient characteristics. For instance, hormonal differences may modulate vitamin D metabolism, with estrogens increasing conversion to its active form, potentially explaining sex-based differences in vitamin D response [92, 93]. Women, particularly postmenopausal, may benefit more in terms of bone and cardiovascular health [94]. In patients with diabetes, vitamin D may improve insulin sensitivity and β-cell function [95, 96, 97], although results from meta-analyses remain mixed [98, 99]. One placebo-controlled trial in South Asian women with insulin resistance showed improved glycemic indices following vitamin D supplementation [100]. Similarly, individuals with advanced CKD often show severe 25(OH)D deficiency, and observational data support a survival benefit from vitamin D analogs in hemodialysis patients [101, 102, 103, 104]. These findings emphasize the need for future RCTs to stratify by baseline vitamin D status, comorbidities, and demographic variables to clarify population-specific benefits.

## 6. Additional Evidence From Observational Studies

To complement interventional evidence, several high-quality observational studies have consistently reported an inverse association between serum 25(OH)D levels and cardiovascular outcomes. Large prospective cohorts such as the Third National Health and Nutrition Examination Survey (NHANES III) [105], the Ludwigshafen Risk and Cardiovascular Health Study (LURIC) study [106], and the Framingham Offspring Study [9] demonstrated that individuals with lower 25(OH)D concentrations had significantly higher risks of all-cause or cardiovascular mortality. Specifically, Melamed *et al*. [105] found that the lowest quartile of 25(OH)D (<17.8 ng/mL) was associated with increased all-cause mortality (hazard ratio (HR) 1.26; 95% CI 1.08–1.46), although cardiovascular-specific associations were not statistically significant. Dobnig *et al*. [106] reported that both the lowest and second-lowest quartiles of 25(OH)D were linked to increased all-cause (HR up to 2.08; 95% CI 1.60–2.70) and cardiovascular mortality (HR up to 2.22; 95% CI 1.57–3.15). Wang *et al*. [9] demonstrated that 25(OH)D levels <15 ng/mL were associated with an increased risk of first cardiovascular events (HR 1.62; 95% CI 1.11–2.36), with an even stronger association observed in hypertensive patients (HR 2.13; 95% CI 1.30–3.48). Other studies provided additional insights into specific cardiovascular outcomes. Pilz *et al*. [107] found that severe vitamin D deficiency (<25 nmol/L) significantly increased the risk of death due to HF (HR 2.84; 95% CI 1.20–6.74) and sudden cardiac death (HR 5.05; 95% CI 2.13–11.97). In a retrospective case-control study of over 20,000 U.S. Veterans [108], patients who achieved 25(OH)D levels ≥75 nmol/L after supplementation had a lower risk of myocardial infarction compared to those who remained <50 nmol/L (OR 0.73; 95% CI 0.55–0.96). Achieving 50–75 nmol/L also reduced MI risk, though to a lesser extent (HR 0.65; 95% CI 0.49–0.85). Other studies linked vitamin D deficiency to neurologic outcomes and coronary disease severity. In ischemic stroke patients [109], 25(OH)D deficiency (<20 ng/mL) was associated greater stroke severity, measured using the National Institutes of Health Stroke Scale (inverse correlation r = –0.408; *p* < 0.001). Finally, in patients undergoing coronary angiography for stable angina [110], 25(OH)D level <20 ng/mL was independently associated with higher Synergy Between Percutaneous Coronary Intervention with TAXUS and Cardiac Surgery (SYNTAX) scores, indicating more severe coronary artery disease (adjusted OR = 0.809 per 1 ng/mL increase; 95% CI 0.743–0.881; *p* < 0.001), with a strong inverse correlation (r = –0.77; *p* < 0.001). These findings, summarized in Table 2 (Ref. [9, 105, 106, 107, 108, 109, 110]), support the hypothesis that low vitamin D status is not only a marker of increased cardiovascular risk but may also represent a potentially modifiable risk factor.

**Table 2.** **Recent observational studies evaluating the association between vitamin D status and cardiovascular outcomes**.

First Author [Reference]	Year	Population (N)	Study design	Outcomes
Melamed, ML [105]	2008	13,331 US adults (NHANES III)	Prospective	Lowest quartile of 25(OH)D (<17.8 ng/mL) associated with increased all-cause mortality (HR 1.26; 95% CI 1.08–1.46); association with CV mortality not statistically significant.
Dobnig, H [106]	2008	3258 patients referred to coronary angiography (LURIC cohort)	Prospective	Lowest quartiles of 25(OH)D (medians 7.6 & 13.3 ng/mL) associated with increased all-cause mortality (HR up to 2.08; 95% CI 1.60–2.70) and cardiovascular mortality (HR up to 2.22; 95% CI 1.57–3.13).
Wang, TJ [9]	2008	1739 participants from the Framingham Offspring Study, free of cardiovascular disease at baseline	Prospective	Lowest 25(OH)D levels (<15 ng/mL) associated with increased risk of first cardiovascular events (HR 1.62; 95% CI 1.11–2.36); association stronger in hypertensive patients (HR 2.13; 95% CI 1.30–3.48).
Pilz, S [107]	2008	3299 patients referred for coronary angiography	Cross-sectional with longitudinal follow-up	Severe vitamin D deficiency (<25 nmol/L) was associated with increased risk of death due to heart failure (HR 2.84; 95% CI 1.20–6.74) and sudden cardiac death (HR 5.05; 95% CI 2.13–11.97).
Acharya, P [108]	2021	20,025 U.S. Veterans with baseline 25(OH)D <50 nmol/L	Retrospective, case-control	Patients who achieved >75 nmol/L after supplementation had lower MI risk compared with those remaining <50 nmol/L (HR 0.73; 95% CI 0.55–0.96); those achieving 50–75 nmol/L also had reduced MI risk (HR 0.65; 95% CI 0.49–0.85).
Simon, J [109]	2024	86 acute ischemic stroke patients	Prospective	25(OH)D deficiency (<20 ng/mL) significantly associated with greater stroke severity (higher NIHSS); inverse correlation (r = –0.408; β = –0.3994; *p * < 0.001).
Candemir, B [110]	2025	120 obese patients (BMI >30 kg/m^2^) undergoing coronary angiography (for stable angina)	Retrospective	Lowest 25(OH)D levels (<20 ng/mL) significantly associated with higher SYNTAX scores (independent predictor: OR = 0.809 per 1 ng/mL increase; 95% CI 0.743–0.881; *p * < 0.001); strong inverse correlation (r = –0.77; *p * < 0.001).

Studies are ordered by year of publication. Abbreviations: BMI, body mass index; CI, confidence interval; CV, cardiovascular; HR, hazard ratio; MI, Myocardial Infarction; LURIC, Ludwigshafen Risk and Cardiovascular Health Study; NHANES III, Third National Health and Nutrition Examination Survey (1988–1994); NIHSS, National Institutes of Health Stroke Scale; SYNTAX, Synergy Between PCI With TAXUS and Cardiac Surgery.

## 7. Recently Completed Trials, Ongoing Research and Future Direction

Several high-quality trials have recently been completed and contribute valuable insights into the cardiovascular effects of vitamin D supplementation (Table 3, Ref. [111, 112, 113, 114, 115]). The VITAL trial [111], which enrolled over 25,000 U.S. adults without prior cardiovascular disease, showed that daily supplementation with 2000 IU of vitamin D_3_ did not reduce the incidence of major cardiovascular events (myocardial infarction, stroke, cardiovascular mortality) compared to placebo. The VITAL Rhythm substudy [112] focused on atrial fibrillation and similarly found no overall reduction in AF incidence, although a subgroup analysis suggested a possible benefit in Black participants. The VITAL Heart Failure (VITAL HF) substudy [116], which evaluated HF outcomes, reported no significant effect of vitamin D_3_ on incident HF. Other recently completed studies include: the DO-HEALTH trial [113], which found no benefit on major adverse cardiovascular events (MACE) or hypertension, although omega-3 supplementation improved lipid profiles; the D2d trial [114], which showed no significant reduction in MACE, but a small improvement in atherosclerotic cardiovascular disease (ASCVD) risk score; the D-Health Trial [115], which reported no reduction in CVD incidence or mortality with monthly high-dose vitamin D_3_, but observed that baseline vitamin D deficiency was associated with higher cardiovascular risk. Finally, the COSMOS trial (NCT02422745) explored cardiovascular outcomes using a factorial design involving cocoa extract and multivitamins; although completed, cardiovascular-specific results are still under analysis. These trials underscore the current limitations of vitamin D interventional research: despite strong mechanistic and observational data, large RCTs have yet to confirm clear cardiovascular benefits. One possible explanation lies in the heterogeneity of study designs, including substantial variability in baseline vitamin D status, dosing regimens (daily vs. bolus), treatment duration (weeks vs. years), and inconsistent thresholds for what constitutes sufficiency or deficiency. In many cases, participants were enrolled regardless of their vitamin D levels, potentially diluting the benefit among those who were already replete. Most trials did not stratify or tailor therapy based on vitamin D deficiency, nor did they incorporate biomarkers to identify those most likely to benefit. For example, individuals with severe deficiency (<10 ng/mL) may experience different physiological responses than those with mild insufficiency, yet this distinction was often overlooked. Furthermore, genetic variability—including polymorphisms in DBP, VDR receptors, and enzymes involved in vitamin D metabolism—remains poorly accounted for in most studies, despite growing evidence that these factors significantly influence absorption, transport, and biological activity. Similarly, metabolic heterogeneity, including comorbid conditions such as chronic kidney disease, obesity, or diabetes, may alter vitamin D kinetics and modify cardiovascular risk independently. Yet, subgroup analyses for these populations remain limited or underpowered in most trials. The lack of consensus on appropriate surrogate endpoints—such as inflammatory markers, left ventricular function, or vascular stiffness—further complicates the interpretation of results and hinders the identification of mechanistic signals that could precede clinical benefit. In short, the “one-size-fits-all” approach in these RCTs may have masked benefits in more vulnerable subgroups, highlighting the urgent need for more personalized, biomarker-guided, and hypothesis-driven trial designs moving forward.

**Table 3.** **Recently completed and ongoing studies investigating vitamin D in cardiovascular diseases**.

Study	ClinicalTrials.gov Identifier or Reference	Start year	Study design	Population	Intervention/Exposure	Primary endpoint(s)	Secondary endpoint(s)	Major findings
*Recently Completed*								
VITAL	[111]	2010	Interventional; Randomized, placebo-controlled trial	Adults aged ≥50 years (men) and ≥55 years (women) without prior CVD, stroke or cancer	Daily vitamin D3 (2000 IU) and/or marine omega-3 fatty acids (EPA 465 mg + DHA 375 mg) vs. placebo for a median of 5.3 years	Major cardiovascular events (myocardial infarction, stroke, cardiovascular mortality), invasive cancer	Coronary revascularization, death from invasive cancer, death from any cause	No significant reduction in the incidence of major cardiovascular events (MI, stroke, CV death) or cancer with vitamin D_3_ (2000 IU/day) supplementation vs. placebo in the general population.
VITAL Rhythm	[112]	2012	Interventional, Randomized, double-blind, placebo-controlled trial	Adults aged ≥50 years (men) and ≥55 years (women) without prior AF, CVD, or cancer (n = 25,871)	Daily vitamin D3 (2000 IU) and/or marine omega-3 fatty acids (EPA 460 mg + DHA 380 mg) vs. placebo for a median of 5.3 years	Incident AF	AF subtypes (paroxysmal vs. persistent AF); sudden cardiac death; ECG changes	No reduction in incident atrial fibrillation with vitamin D_3_ (2000 IU/day) or omega-3 fatty acids.
DO-HEALTH	[113]	2012	Randomized, placebo-controlled trial	Elderly (mean age 74), 61.7% women	Vitamin D3 (2000 IU/day) ± omega-3 ± SHEP vs. placebo	Hypertension, MACE, lipid profile	Lipid biomarkers, BP, physical activity	No benefit on MACE; omega-3 improved lipids
D2d	[114]	2013	Randomized, placebo-controlled trial	Adults with prediabetes (n = 2423)	Vitamin D3 (4000 IU/day) vs. placebo	MACE, ASCVD risk score	BP, lipids, hs-CRP, ASCVD risk factors	No MACE reduction; small benefit in ASCVD risk score
D-Health Trial	[115]	2014	Randomized, placebo-controlled trial	21,315 adults aged 60–84 years in Australia, without known vitamin D deficiency	Monthly oral vitamin D3 (60,000 IU) vs. placebo for 5 years	CVD incidence and mortality	All-cause mortality	No CVD reduction; deficiency linked to higher CVD risk
*Ongoing*								
TARGET-D	NCT02996721	2017	Interventional; Randomized, Open-Label, Parallel Assignment	Patients with a history of MI and vitamin D deficiency	Standard of care vs. individualized vitamin D3 supplementation to achieve 25(OH)D >40 ng/mL	Death, myocardial infarction, heart failure hospitalization, and CVA	NA	Pending
INVITE	NCT02925195	2017	Interventional; Randomized, Double-blind, Parallel Assignment, placebo-controlled trial	1600 Adults from the Multi-Ethnic Study of Atherosclerosis (MESA) study	Daily vitamin D3 (2000 IU) vs. placebo in 3:1 ratio for 16 weeks	To identify genetic polymorphisms, clinical characteristics, and biomarkers that modify the biologic response to vitamin D3 treatment	Change in blood pressure, in urine calcium concentrations and serum calcium concentrations	Pending
VINDICATE-MI	NCT03086746	2018	Prospective cohort	Adults (≥18 years) with recent (<72 hours) STEMI	Baseline vitamin D levels	Left ventricular remodeling (≥5% reduction in LVEF or ≥15% increase in LVESVi) at 6 months	Vitamin D, Vitamin D binding protein and PTH levels	Pending
					Vitamin D3 supplementation (4000 IU daily) vs. placebo			
VINDICATE 2	NCT03416361	2023	Interventional; Randomized, Quadruple-Blind, Parallel Assignment	Adults (≥18 years) with CHF due to LVSD (LVEF <50%), vitamin D deficiency (<50 nmol/L), and at least one high-risk criterion (recent HF hospitalization, high-dose diuretics, diabetes, or ischemic heart disease)	4000 IU Vitamin D3 (chewable tablets, 2 per day) vs. placebo	Time to death or first hospitalization for heart failure (24 months)	Total mortality, cost-effectiveness (ICER for vitamin D), change in patient quality of life (EQ5D-5L)	Pending

Studies are ordered by year of publication. Abbreviations: AF, Atrial fibrillation; ASCVD, Atherosclerotic Cardiovascular Disease; BP, Blood Pressure; CHF, Chronic heart failure; CVA, Cerebrovascular accident; CVD, Cardiovascular disease; DHA, Docosahexaenoic acid; ECG, Electrocardiogram; EQ5D-5L, EuroQol 5-Dimension 5-Level questionnaire (measure of health-related quality of life); EPA, Eicosapentaenoic acid; HF, Heart failure; hs-CRP, High-Sensitivity C-Reactive Protein; ICER, Incremental cost-effectiveness ratio; LVEF, Left ventricular ejection fraction; LVESVi, Left ventricular end-systolic volume index; LVSD, Left ventricular systolic dysfunction; MACE, Major Adverse Cardiovascular Events; MI, Myocardial infarction; NA, Not Applicable; SHEP, simple home-based exercise program; RCT, Randomized Controlled Trial; STEMI, ST-elevation myocardial infarction.

To address these gaps, several ongoing studies are focusing on more personalized and targeted approaches. Trials such as INVITe (NCT02925195) are ongoing and aim to uncover genetic and metabolic predictors of individual responses to vitamin D. Others, like TARGET-D (NCT02996721) and VINDICATE 2 (NCT03416361), are selectively enrolling participants with documented deficiency and high cardiovascular risk, aiming to clarify whether supplementation is beneficial in those who are most likely to respond. Pediatric populations are also being investigated. The Vitamin D and Vascular Health in Children (NCT01797302) trial assesses vascular function in obese children and evaluates the effects of daily supplementation (600–2000 IU) over six months, while Low vs. Moderate to High Dose Vitamin D for Prevention of COVID-19 (NCT04868903) explores optimal dosing in infants. In acute care settings, the VIOLET trial (NCT03096314) tests whether a single high dose (540,000 IU) of vitamin D_3_ could reduce mortality in critically ill, vitamin D–deficient patients. Additional studies are exploring metabolic and structural outcomes, such as glycemic control in children with type 1 diabetes (NCT05141968) and cardiac remodeling following myocardial infarction or in HF, such as in VINDICATE-MI (NCT03086746). Collectively, these trials aim to address key knowledge gaps regarding optimal dosing strategies, the most responsive target populations, and the true efficacy of vitamin D in cardiovascular prevention and therapy. Their results may help reconcile the current discrepancies between observational and interventional evidence and determine whether vitamin D can play a meaningful role in cardiovascular health.

## 8. Conclusion

Vitamin D remains a compelling yet enigmatic player in cardiovascular health. The Good includes its anti-inflammatory, antifibrotic, and vasoprotective properties. The Bad highlights concerns surrounding the potential adverse effects of over-supplementation and the unmet expectations in large RCTs. Finally, the Unknown lies in the persistent gap between association and causation, complicated by confounding variables, heterogeneous populations, and inconsistencies in dosing regimens. As ongoing large-scale trials unfold, there is cautious optimism that great clarity will emerge, revealing whether vitamin D is a silent bystander or a modifiable contributor to cardiovascular disease prevention and management.

## Abbreviations

VDR, vitamin D receptor; DBP, vitamin D binding protein; PTH, parathyroid hormone; FGF-23, fibroblast growth factor-23; RXR, retinoid X receptor; ROS, reactive oxygen species; COX-1, cyclooxygenase-1; IL, interleukin; TNF-α, tumor necrosis factor-alpha; TLR, Toll-like receptor; CRP, C-reactive protein; LDL, low-density lipoprotein; MMP, matrix metalloproteinase; RCT, randomized controlled trial; CVD, cardiovascular disease; CKD, chronic kidney disease; OR, odds ratio; IOM, Institute of Medicine; VINDICATE, VItamiN D treatIng Patients with Chronic heArT failurE; VITAL, VITamin D and omegA-3 TriaL; VIDA, Vitamin D Assessment Study; INVITe, Individual response to Vitamin D trial; COSMOS, COcoa Supplement and Multivitamin Outcomes Study; VIOLET, Vitamin D to Improve Outcomes by Leveraging Early Treatment; TARGET-D, Trial of Administration of Vitamin D after Myocardial Infarction; OR, odds ratio; CI, confidence interval; HR, hazard ratio; MACE, major adverse cardiovascular events; ASCVD, atherosclerotic cardiovascular disease.

## Declaration of AI and AI-Assisted Technologies in the Writing Process

This manuscript was prepared with the assistance of artificial intelligence tools, including ChatGPT-4o (OpenAI, San Francisco, CA, USA) and Grammarly (Grammarly Inc., San Francisco, CA, USA), following established best practices (Biondi-Zoccai G, editor. ChatGPT for Medical Research. Torino: Edizioni Minerva Medica; 2024). Figures were created using BioRender.com. The authors have thoroughly reviewed, edited, and approved the final content, and they take full responsibility for the accuracy, integrity, and intellectual contributions of the work. All ethical standards and guidelines governing the use of artificial intelligence in research have been fully respected.
